# Supplementary material for: Ecology and Genetic Structure of the Parasitoid Phobocampe confusa (Hymenoptera: Ichneumonidae) in Relation to Its Hosts, Aglais Species (Lepidoptera: Nymphalidae)
Source: Insects. 2020 Jul 28;11(8):478. doi: 10.3390/insects11080478 (PMC7469161; doi:10.3390/insects11080478)
Supplement: Supplementary file 1 [file insects-11-00478-s001.pdf]

Supplementary Material 1: Climatic variations between years and counties (Sweden)

Temperature data were acquired throughout the field sampling with tinytag recorders (Tinytag Plus 2, TGP-4020) placed in the field in nettle patches at a height between 50 and 80 cm. We tested for the differences between counties and years in growing degree day-base 13°C (GDD13) accumulated over the reproductive season (from earliest May 9th to latest August 29th). For that, we modelled GDD13 using a generalized additive model with a normal error distribution including year, county and the interaction between year and county as linear effects, and the Julian day as a non-linear effect. We observed significant differences in GDD13 accumulated over the reproductive season between counties ($F = 134.9$, $p < 0.001$) and years ($F = 2333.5$, $p < 0.001$, Fig. S1). Between years, the relative change in the GDD13 accumulated was highest in the counties of Skåne and Kronoberg (estimate = 131.2, $t = 48.3$, $p < 0.001$ and estimate = 17.4, $t = 5.1$, $p < 0.001$, in Kronoberg and Skåne, respectively) than in the county of Stockholm (estimate = -37.3, $t = 3.7$, $p < 0.001$) (Fig. S1). In Skåne, GDD13 at the end of the reproductive season was of 217.49 ± 14.41°C in 2017 and of 531.13 ± 35.28°C in 2018. In Kronoberg, GDD13 at the end of the reproductive season was of 155.24 ± 9.27 °C in 2017 and of 427.65 ± 35.83°C in 2018. In Stockholm, GDD13 at the end of the reproductive season was of 260.78 ± 24.96°C in 2017 and of 518.70 ± 30.91°C in 2018.

Precipitation data were extracted for each site from the E-OBS v19.0e ([1], https://www.ecad.eu/). The resolution of these data is of 0.1 degree, which is about 11.11 km. We extracted these data in R 3.6.1 [2], using the packages ncdf4, raster, rgdal, sf, and lubridate [3–7]. Here, we only considered precipitations during the reproductive season of our study species; that is, precipitation from May 1st to August 31st. We modelled cumulative precipitation using a generalized additive model with a normal error distribution including year, county and the interaction between year and county as linear effects, and the Julian day as a non-linear effect. The cumulative precipitation was log-transformed prior to inclusion in the model. We observed significant differences in cumulative precipitation over the reproductive season between counties ($F = 284.3$, $p < 0.001$) and years ($F = 7.8$, $p = 0.005$, Fig. S1). Between years, the relative change in cumulative precipitation was higher in the counties of Skåne and Stockholm (estimate = -0.91, $t = -23.29$, $p < 0.001$ and estimate = -0.42, $t = -10.52$, $p < 0.001$, in Skåne and Stockholm, respectively) than in the county of Kronoberg, which showed the least change in precipitation profile between years (estimate = -0.09, $t = -2.80$, $p = 0.005$) (Fig. S1).

Thus, associated with the overall increase in average temperature over the season, the precipitation was significantly lower in 2018 compared to 2017. 2018 was an abnormally dry year with respect to 1981-2010 (https://climate.copernicus.eu/dry-and-warm-spring-and-summer).

The between years variation in temperature profile is correlated to the observed change in phenological overlap between years. Following the same procedure as described in the manuscript section 2.3., and replacing the variable year by GDD13, model selection procedure resulted in the same best model (region: $F = 0.005$, $p = 0.94$; GDD13: $F = 4.75$, $p = 0.03$; host: $F = 6.19$, $p = 0.004$; region x host: $F = 6.96$, $p = 0.01$). The explained variance of this model is comparable, of 24.2%.

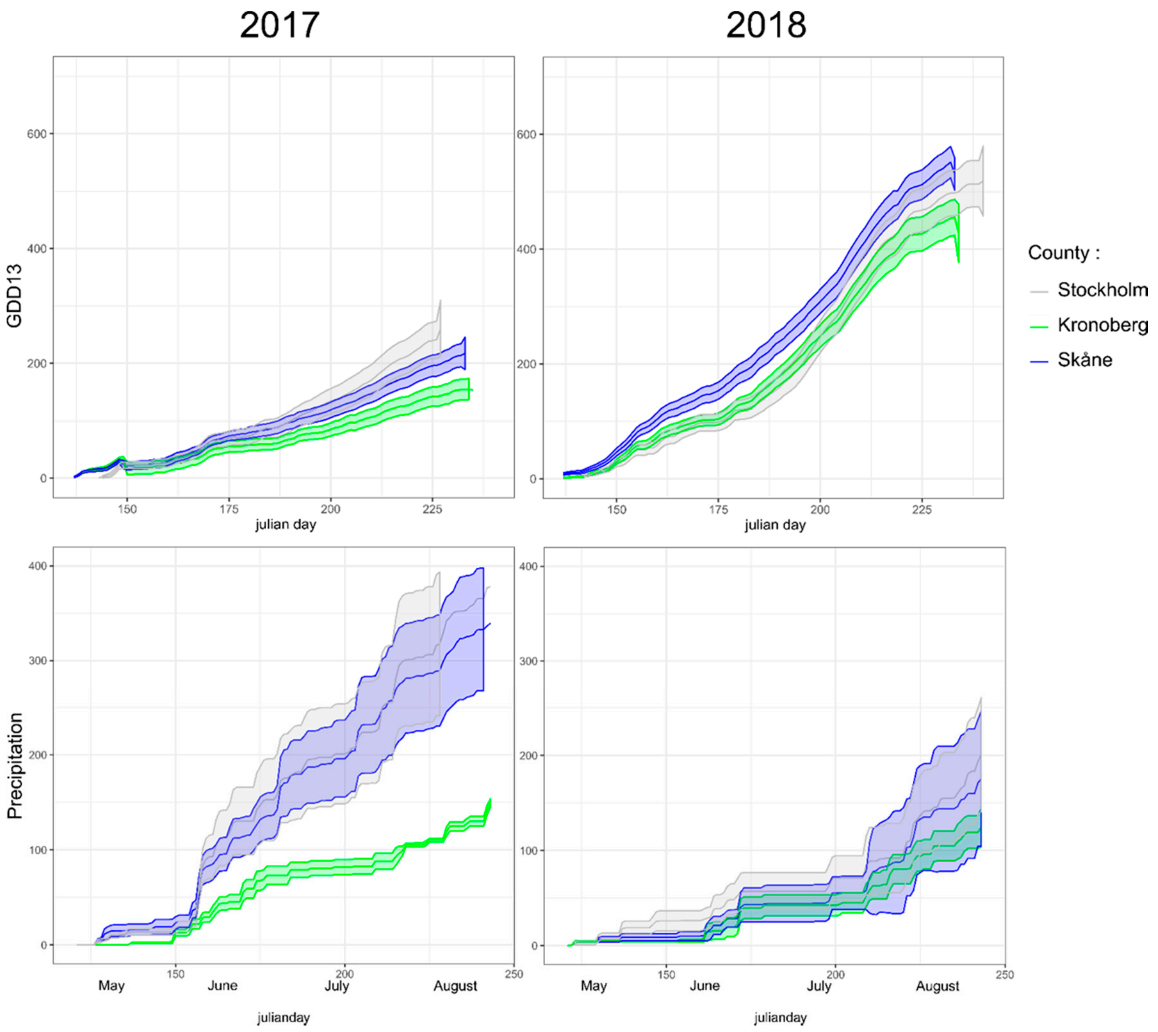

Figure S1. Cumulative growing degrees-days above 13°C and precipitation in Kronoberg, Skåne, and Stockholm in 2017 and 2018.

Acknowledgments: We acknowledge the E-OBS dataset from the EU-FP6 project UERRA (http://www.uerra.eu) and the Copernicus Climate Change Service, and the data providers in the ECA&D project (https://www.ecad.eu). AH acknowledges support from the Swedish Research Council (2016-06737).

Reference

1. Cornes, R.C.; van der Schrier, G.; van den Besselaar, E.J.M.; Jones, P.D. An Ensemble Version of the E-OBS Temperature and Precipitation Data Sets. *Journal of Geophysical Research: Atmospheres* **2018**, 123, 9391–9409, doi:10.1029/2017JD028200.
2. R Core Team R: A Language and Environment for Statistical Computing; R Foundation for Statistical Computing: Vienna, Austria, 2020.
3. Pierce, D. ncdf4: Interface to Unidata netCDF (Version 4 or Earlier) Format Data Files. R package version 1.17, 2019.
4. Hijmans, R.J. raster: Geographic Data Analysis and Modeling. R package version 3.0-12, 2020.
5. Bivand, R.; Keitt, T.; Rowlingson, B. rgdal: Bindings for the "Geospatial" Data Abstraction Library. R package version 1.4-8, 2019.
6. Pebesma, E. Simple features for R: standardized support for spatial vector data. *The R Journal*, **2018**, 10, 439–446.
7. Grolemund, G.; Wickham, H. Dates and Times Made Easy with lubridate. *Journal of Statistical Software* **2011**, 40, doi:10.18637/jss.v040.i03.

Supplementary Material 2: Phenology and temporal window of attack of the hosts (*Aglais urticae* and *A. io*) by *Phobocampe confusa*.

Parasitism by *Phobocampe confusa* started mid-May, both in the south and the north of Sweden and in both years of our study (2017 and 2018). In 2017, cases of parasitism by *P. confusa* were found until mid-July in the north and until the beginning of August in the south. The time window of the occurrence of *P. confusa* was substantially shorter in 2018 with the last occurrence of the species being recorded 4 and 6 weeks earlier in the northern and the southern regions, respectively (year = 1.81, t = -1.75, p = 0.092). The reduction in the time window of occurrence of *P. confusa* was most pronounced in the north (average time widow across sites in the north ± se: 2017 = 6.0 ± 1.10 weeks, 2018 = 2.0 ± 1.15 weeks) compared to the south (average time widow across sites in the south ± se: 2017 = 5.0 ± 3.70 weeks, 2018 = 4.33 ± 2.65 weeks), even though the difference between regions was not significant. Note also that the phenology of *A. urticae* and *A. io* started and ended earlier in 2018 than in 2017 in the north (Table S1). In the south, the time windows during which the native species were collected in 2017 and 2018 are comparable (Table S1). This shift in the phenology of the butterflies in the north could also explain the substantial decrease in the number of larvae parasitized by *P. confusa*.

P. confusa emerged from *A. urticae* larvae collected from the 2 to 5th instar. We detected no evidence of *P. confusa* parasitism on first instar larvae of *A. urticae* (that is 51 larvae collected across 8 nests). Thus, the temporal window of attack of *P. confusa* for this host corresponds to the time during which *A. urticae* develops from 2nd to 4th instar. From larvae monitored in our laboratory rearing conditions (23°C and 22L:2D light regime), this time is on average 5.15 days. This measure of development time is most certainly longer in the field, the mean temperature being lower, and therefore should be taken as a relative measure.

P. confusa was found to emerge from *A. io* larvae collected from 2nd to 5th instar and from 5 larvae from one nest collected at the first instar (out of 32 larvae collected across 6 nests). The temporal window of attack of *P. confusa* for this host, again with respect to our laboratory conditions (23°C and 22L:2D light regime), is on average 7.80 days.

Parasitism rate was highest when larvae were collected at the 4th instar. On the other hand, parasitism rate for larvae collected at the fifth instar was significantly lower, mainly because *P. confusa* often emerges from the body of its host already at the 4th instar. Moreover, a larger proportion of *P. confusa* emerged from 4th instar larvae in *A. io* than in *A. urticae.* This difference is probably related to the difference in the development between the two butterfly species. The pupation time in *A. io* is longer than in *A. urticae* and the larvae reach a larger size, which probably explains why the parasitoid reaches maturity at an earlier larval development stage in *A. io* than in *A. urticae*.

Table S1. showing the first and last week of occurrence of *P. confusa* and each host butterfly, according to region and year. Weeks are expressed in numbers. In 2017, week 21 started May 22nd, in 2018, week 20 started May 14th.

Species	Year / region	2017		2018	
		1st week	Last week	1st week	Last week
P. confusa	North	21	29	21	25
	South	20	32	20	26
A. urticae	North	21	33	19	29
	South	20	32	18	32
A. io	North	25	29	23	27
	South	24	30	22	32
A. levana	North	-	-	-	-
	South	24	34	22	34

Supplementary Material 3: Habitat characteristics associated with *Phobocampe confusa* occurrence for buffer zone radii varying from 10 to 500m.

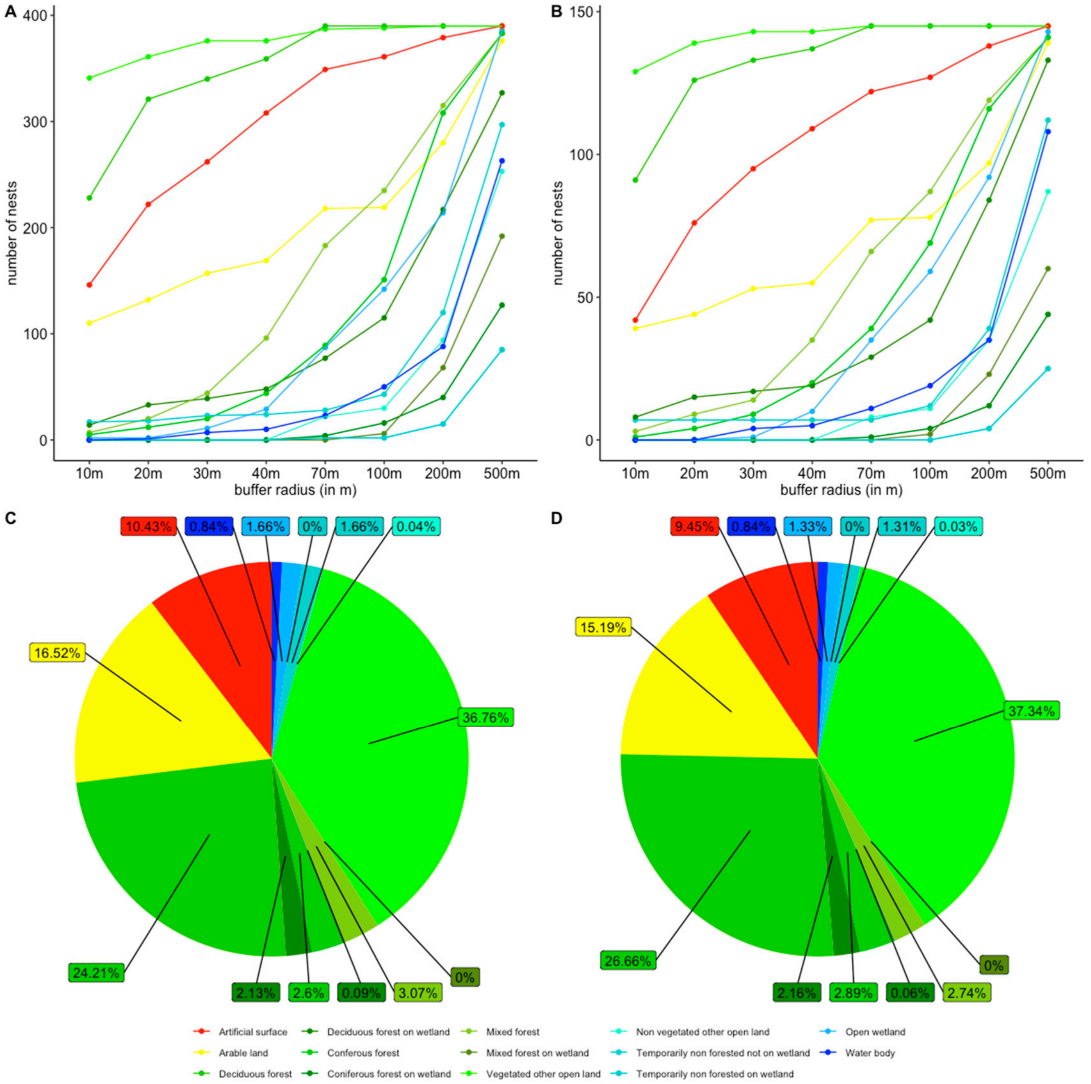

Figure S2. Number of butterfly nests sampled surrounded by each type of land use and for each radius of the buffer zone considered (from 10m to 500m radius) for (A) all butterfly nests sampled within the phenological window of occurrence of *P. confusa* (n = 390) and (B) for the subset of butterfly nests parasitized by *P. confusa* (n = 145). In (C) and (D), pie charts representing the average land use composition within a 100m radius of the butterfly nests sampled, for all butterfly nests sampled within the phenological window of occurrence of *P. confusa* (C) and for the subset of butterfly nests parasitized by *P. confusa* (D).

insects

MDPI

Table S2. summary of the models built to examine the impact of the land use heterogeneity and fragmentation of the habitat on the propensity of a butterfly nest to be parasitized by *P. confusa*. We built one model per buffer zones considered (10, 20, 30, 40, 70, 100, 200 and 500m radius) in order to examine the impact of land use at different distance around each nest. The habitat variables selected in the final model are framed in red.

Buffers size	Parameters	Intercept	Overlap	Overlap2	3rd instar	4th instar	5th instar	Artificial surface (%)	Length of edges (m)	Deciduous forest (%)
10 m	estimate ± se	- 4.74 ± 0.80	10.61 ± 2.60	-6.48 ± 2.28	0.82 ± 0.37	1.28 ± 0.34	-0.10 ± 0.31	-0.015 ± 0.007	0.015 ± 0.009	-
n = 390	z value	-5.91	4.09	-2.23	2.23	3.80	-0.32	-2.07	1.67	-
AIC = 429.3	p	<0.001	<0.001	0.004	0.026	<0.001	0.75	0.039	0.095	-
20 m	estimate ± se	- 4.92 ± 0.84	10.90 ± 2.61	-6.77 ± 2.28	0.83 ± 0.37	1.34 ± 0.34	-0.090 ± 0.313	-0.026 ± 0.009	0.008 ± 0.004	-
n = 390	z value	-5.86	4.18	-2.97	2.22	3.94	-0.29	-2.97	2.06	-
AIC = 425.0	p	<0.001	0.003	0.003	0.026	<0.001	0.77	0.003	0.004	-
30 m	estimate ± se	- 4.26 ± 0.77	10.24 ± 2.57	-6.21 ± 2.25	0.82 ± 0.37	1.30 ± 0.34	-0.113 ± 0.308	-0.016 ± 0.010	-	-
n = 390	z value	-5.50	3.98	-2.76	2.25	3.88	-0.37	-1.69	-	-
AIC = 430.1	p	<0.001	<0.001	0.006	0.024	<0.001	0.71	0.092	-	-
40 m	estimate ± se	- 4.47 ± 0.76	10.30 ± 2.55	-6.25 ± 2.23	0.82 ± 0.36	1.30 ± 0.33	-0.122 ± 0.307	-	-	-
n = 390	z value	-5.86	4.04	-2.80	2.25	3.88	-0.40	-	-	-
AIC = 430.9	p	<0.001	<0.001	0.005	0.025	<0.001	0.69	-	-	-
70 m	estimate ± se	- 4.47 ± 0.76	10.30 ± 2.55	-6.25 ± 2.23	0.82 ± 0.36	1.30 ± 0.33	-0.122 ± 0.307	-	-	-
n = 390	z value	-5.86	4.04	-2.80	2.25	3.88	-0.40	-	-	-
AIC = 430.9	p	<0.001	<0.001	0.005	0.025	<0.001	0.69	-	-	-
100 m	estimate ± se	- 4.10 ± 0.80	11.05 ± 2.66	-7.06 ± 2.33	0.79 ± 0.37	1.29 ± 0.34	-0.14 ± 0.31	-0.047 ± 0.022	-	-
n = 390	z value	-5.13	4.15	-3.03	2.16	3.82	-0.46	-2.17	-	-
AIC = 428.1	p	<0.001	<0.001	0.005	0.031	<0.001	0.65	0.030	-	-
200 m	estimate ± se	- 4.04 ± 0.79	10.81 ± 2.60	-6.82 ± 2.27	0.79 ± 0.37	1.29 ± 0.34	-0.14 ± 0.31	-0.064 ± 0.027	-	-
n= 390	z value	-5.13	4.16	-3.0	2.17	3.81	-0.48	-2.40	-	-
AIC = 426.9	p	<0.001	<0.001	0.003	0.030	<0.001	0.63	0.017	-	-
500 m	estimate ± se	- 4.94 ± 0.81	10.26 ± 2.54	-6.25 ± 2.23	0.84 ± 0.37	1.32 ± 0.34	-0.13 ± 0.31	-	-	0.026 ± 0.014
n = 390	z value	-6.14	4.04	-2.80	2.30	3.92	-0.42	-	-	1.85
AIC = 429.5	p	<0.001	<0.001	0.005	0.022	<0.001	0.68	-	-	0.065

Table S3. Summary of the models built to examine the impact of the land use heterogeneity and fragmentation of the habitat on the intensity of parasitism, that is the proportion of larvae parasitized by *P. confusa*. We built one model per buffer zones considered (10, 20, 30, 40, 70, 100, 200 and 500m radius) in order to examine the impact of land use at different distance around each nest. The habitat variables selected in the final models are framed in red.

Buffer size	parameters	Intercept	Year (2018)	Week	Week^2	Overlap	Overlap^2	*A. urticae*	3rd instar	4th instar	5th instar	Region (South)	South x 2018	Deciduous forest (%)	Artificial surface (%)	Arable land (%)
10 m n = 145	estimate ± se	- 4.63 ± 0.78	-0.11 ± 0.33	0.67 ± 0.27	-0.084 ± 0.036	6.30 ± 1.84	-5.29 ± 1.57	0.39 ± 0.18	-0.32 ± 0.19	-0.076 ± 0.175	0.16 ± 0.21	-0.24 ± 0.19	0.78 ± 0.33	0.005 ± 0.003	-	0.006 ± 0.002
AIC =	z value	-5.90	-0.35	2.43	-2.31	3.42	-3.38	2.17	-1.66	-0.43	0.75	-1.27	2.36	1.98	-	2.53
542.3	p	<0.001	0.72	0.015	0.021	<0.001	<0.001	0.030	0.097	0.67	0.45	0.20	0.019	0.048	-	0.011
20 m n = 145	estimate ± se	- 5.28 ± 0.74	-0.15 ± 0.31	0.94 ± 0.25	-0.113 ± 0.034	6.22 ± 1.79	-5.28 ± 1.50	0.53 ± 0.17	-	-	-	-0.28 ± 0.18	0.988 ± 0.319	-0.010 ± 0.003	-	-
AIC =	z value	-7.15	-0.48	3.77	-3.29	3.48	-3.52	3.14	-	-	-	-1.60	3.10	3.33	-	-
544.8	p	<0.001	0.63	<0.001	0.001	<0.001	<0.001	0.002	-	-	-	0.11	0.002	<0.001	-	-
30 m n = 145	estimate ± se	- 4.62 ± 0.77	-0.35 ± 0.32	0.67 ± 0.27	-0.082 ± 0.036	6.04 ± 1.78	-5.10 ± 1.50	0.42 ± 0.18	-0.30 ± 0.19	-0.065 ± 0.174	0.16 ± 0.21	-0.30 ± 0.18	1.07 ± 0.32	0.010 ± 0.003	-	-
AIC =	z value	-6.02	-1.10	2.46	-2.27	3.39	-3.40	2.31	-1.59	-0.37	0.76	-1.68	3.34	3.15	-	-
543.52	p	<0.001	0.27	0.014	0.023	<0.001	<0.001	0.021	0.11	0.71	0.45	0.09	0.019	0.002	-	-
40 m n = 145	estimate ± se	- 4.80 ± 0.77	-0.36 ± 0.32	0.69 ± 0.28	-0.084 ± 0.037	5.67 ± 1.78	-4.81 ± 1.50	0.47 ± 0.18	-0.35 ± 0.18	-0.141 ± 0.177	0.11 ± 0.21	-0.35 ± 0.18	1.14 ± 0.32	0.015 ± 0.004	0.015 ± 0.006	-
AIC =	z value	-6.23	-1.11	2.50	-2.27	3.19	-3.21	2.54	-1.85	-0.80	0.55	-2.00	3.52	3.91	2.31	-
539.4	p	<0.001	0.27	0.012	0.023	0.001	0.001	0.011	0.065	0.43	0.58	0.046	<0.001	<0.001	0.021	-
70 m n = 145	estimate ± se	- 4.80 ± 0.77	-0.35 ± 0.32	0.71 ± 0.28	-0.082 ± 0.037	5.40 ± 1.77	-4.64 ± 1.50	0.46 ± 0.18	-0.397 ± 0.192	-0.168 ± 0.179	0.073 ± 0.209	-0.45 ± 0.18	1.09 ± 0.32	0.019 ± 0.005	0.021 ± 0.009	-
AIC =	z value	-6.20	-1.08	2.56	-2.24	3.05	-3.10	2.51	-2.06	-0.94	0.35	-2.58	3.38	3.55	2.32	-
542.4	p	<0.001	0.28	0.010	0.025	0.002	0.002	0.012	0.039	0.347	0.73	0.010	<0.001	<0.001	0.021	-
100 m	estimate ± se	- 4.46 ± 0.76	-0.36 ± 0.32	0.66 ± 0.28	-0.075 ± 0.037	4.44 ± 1.76	-3.80 ± 1.49	0.43 ± 0.18	-0.436 ± 0.194	-0.170 ± 0.180	0.052 ± 0.221	-0.52 ± 0.18	1.07 ± 0.32	0.018 ± 0.005	-	-
n = 145	z value	-5.88	-1.11	2.36	-2.01	2.52	-2.55	2.38	-2.25	-0.95	0.25	-2.92	3.32	3.39	-	-
AIC = 542.7	p	<0.001	0.27	0.018	0.044	0.012	0.011	0.017	0.025	0.344	0.8	0.004	0.024	<0.001	-	-
200 m	estimate ± se	- 4.46 ± 0.77	-0.18 ± 0.32	0.82 ± 0.29	-0.11 ± 0.04	4.48 ± 1.76	-3.60 ± 1.48	0.47 ± 0.18	-0.42 ± 0.19	-0.182 ± 0.179	0.061 ± 0.211	-0.44 ± 0.18	0.84 ± 0.32	0.015 ± 0.005	-	-
n = 144	z value	-5.80	-0.54	2.78	-2.73	2.54	-2.43	2.59	-2.18	-1.02	0.29	-2.48	2.59	2.77	-	-
AIC = 527.6	p	<0.001	0.59	0.005	0.006	0.011	0.015	0.010	0.029	0.31	0.77	0.013	0.010	0.006	-	-
500 m	estimate ± se	- 4.34 ± 0.76	-0.29 ± 0.32	0.75 ± 0.27	-0.094 ± 0.036	5.40 ± 1.75	-4.47 ± 1.47	0.41 ± 0.18	-0.319 ± 0.191	-0.0009 ± 0.172	0.206 ± 0.204	-0.39 ± 0.17	1.036 ± 0.320	-	-	-
n = 145	z value	-5.70	-0.90	2.76	-2.61	3.09	-3.04	2.27	-1.68	0.005	1.01	-2.28	3.24	-	-	-
AIC = 551.42	p	<0.001	0.37	0.006	0.009	0.002	0.002	0.024	0.094	0.996	0.312	0.023	0.001	-	-	-
